# Arthroscopic capsular release combined with Latarjet procedure for chronic locked anterior shoulder dislocation secondary to epilepsy: a case report with one-year follow-up

**DOI:** 10.3389/fsurg.2026.1852643

**Published:** 2026-07-08

**Authors:** Zeyan Chen, Jiancong Chen, Guoliang Wang, Zhenfeng Zhang

**Affiliations:** Department of Orthopedics, Guangzhou Development District Hospital, Guangzhou, China

**Keywords:** arthroscopy, Bankart lesion, chronic anterior shoulder dislocation, epilepsy, Latarjet procedure

## Abstract

**Background:**

Chronic anterior shoulder dislocation is rare and poses significant therapeutic challenges due to associated structural injuries. This case report details the management of a chronic dislocation with complex pathologies.

**Case presentation:**

A 28-year-old female patient presented with persistent left shoulder deformity and restricted motion 1 year after an anterior dislocation. Imaging revealed a locked anterior dislocation, Bankart lesion, Hill-Sachs lesion, pseudoglenoid formation, and rotator cuff atrophy. Preoperative functional scores were poor.

**Intervention:**

Arthroscopic posterior capsular release and glenoid osteoplasty were performed, followed by open Latarjet procedure with coracoid transfer and screw fixation. Temporary Kirschner wire stabilization was maintained for 2 weeks.

**Outcomes:**

At 1-year follow-up, shoulder mobility significantly improved and functional scores markedly increased with no recurrence. The patient reported high satisfaction.

**Conclusion:**

The Latarjet procedure combined with arthroscopic release effectively restored stability and function in chronic anterior shoulder dislocation caused by epileptic seizure. Early postoperative rehabilitation is critical for optimal recovery.

## Introduction

The shoulder dislocation is the most common type of joint dislocation in the human body (1), It may result from direct or indirect trauma, as well as epileptic seizures (2, 3). Chronic or neglected anterior shoulder dislocation is extremely rare and is often associated with structural abnormalities such as Bankart lesions, Hill-Sachs lesions, significant bone loss, rotator cuff tears, glenohumeral osteoarthritis, and capsular tears (4–6). Chronic locked anterior shoulder dislocation poses a challenging clinical problem for both patients and surgeons, and there is currently no standard treatment protocol (7). Herein, we report the case of a 28-year-old female patient with chronic locked anterior shoulder dislocation secondary to epileptic seizures, complicated by Bankart lesion and Hill-Sachs lesion,which was successfully managed with arthroscopic release and the Latarjet procedure.

## Case report

A 28-year-old female patient presented with persistent left shoulder deformity and restricted range of motion for 1 year. The patient had a high school education and a history of epilepsy. He was treated with levetiracetam tablets and oxcarbazepine tablets. One year before he was admitted to hospital, he suffered from anterior dislocation of the shoulder due to seizure. At that time, he was relieved after taking analgesic drugs himself, so he did not go to the hospital. Soon after, he underwent brain surgery to control epilepsy, and the epilepsy did not recur after operation. However, the patient developed progressive shoulder deformity and dysfunction, no neurological damage, and lost his job. Therefore, the patient was treated in our hospital after a year of dislocation of the left shoulder, and the left shoulder joint was restricted and deformed, with a VAS score of 3.

**Figure 1:**
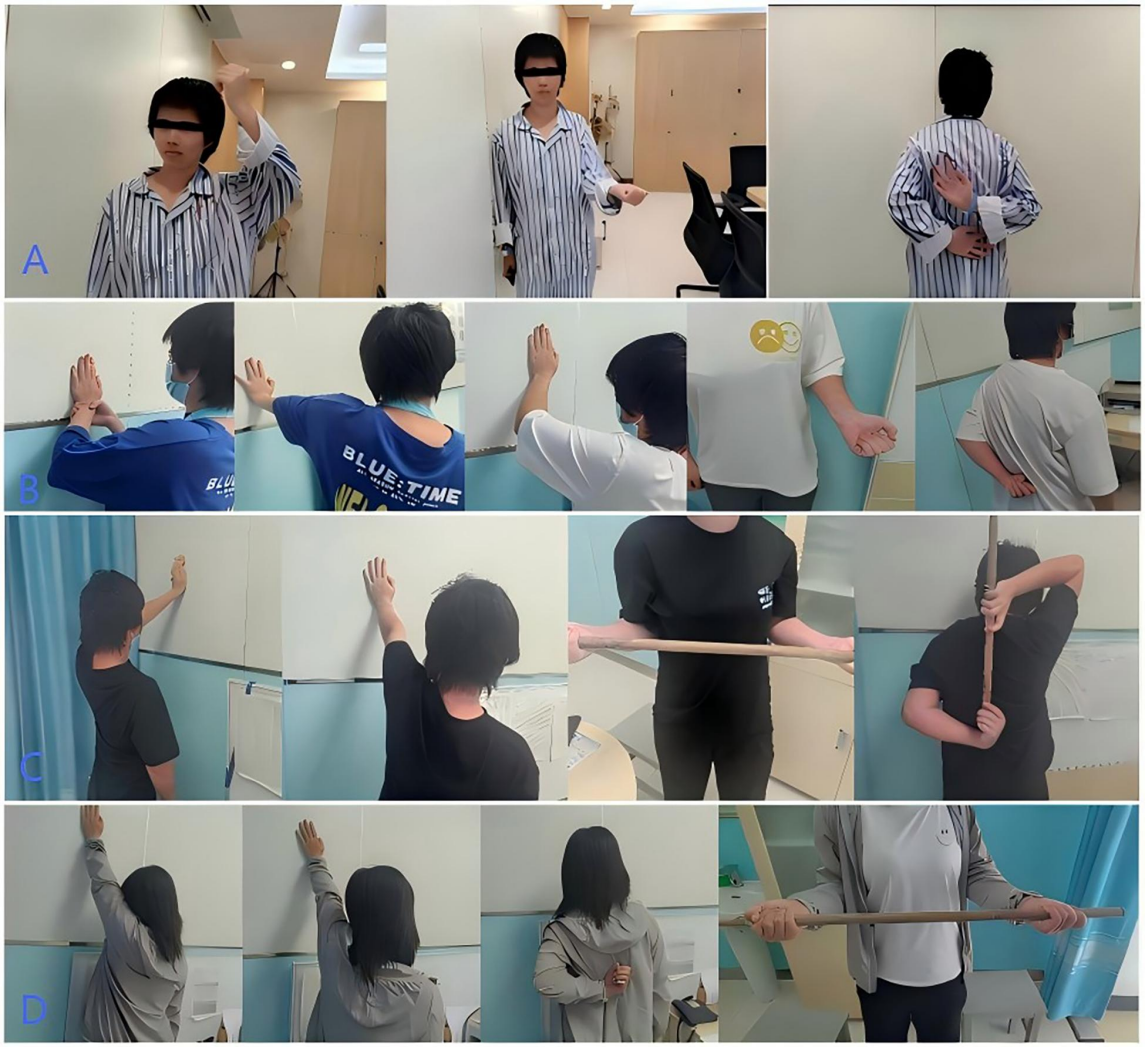
**(A)** Preoperative shoulder range of motion. **(B)** Shoulder range of motion at 3 weeks and 6 weeks postoperatively. **(C)** Shoulder range of motion at 10 weeks postoperatively. **(D)** Shoulder range of motion at one year postoperatively.

**Figure 2:**
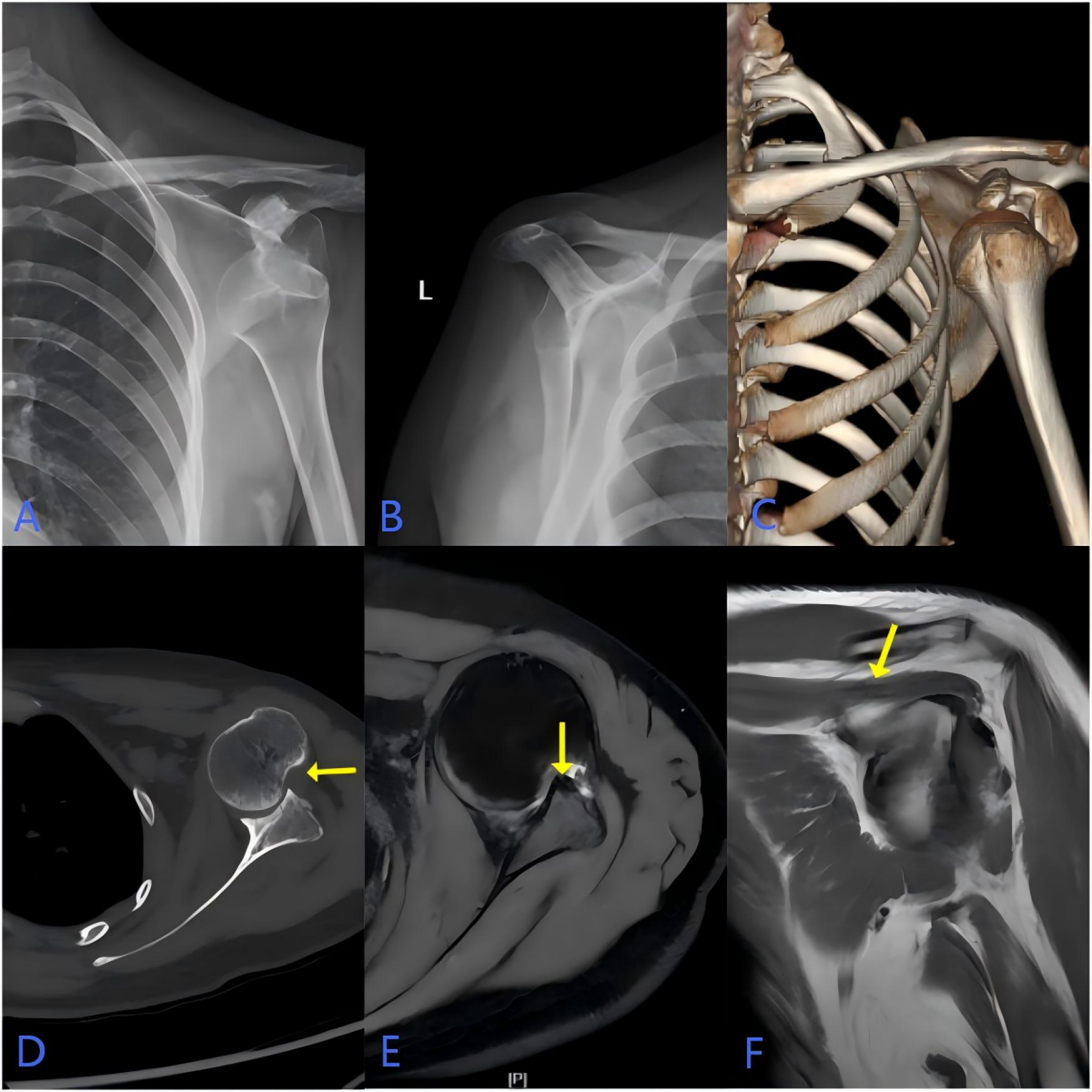
**(A,B)** preoperative x-ray examination revealed an anterior dislocation of the shoulder joint. **(C,D)** Preoperative CT scan images revealed a shoulder joint dislocation and a Hill-Sachs lesion in the humeral head (pointed by yellow arrow). **(E,F)** Preoperative MRI scan revealed a Bankart lesion (pointed by yellow arrow in 2E), and thinning of the rotator cuff tendons (pointed by yellow arrow in 2F).

Physical examination revealed muscle atrophy in the left shoulder, square shoulder deformity, and a globular prominence below the left coracoid process in the supine position. Positive Dugas sign was observed, with both active and passive movements restricted, and no associated vascular or nerve injury in the distal limb. The left shoulder demonstrated limited range of motion: forward flexion 80°, extension 30°, abduction 10°, internal rotation at the side to L3 level (Figure 1A). Preoperative functional assessments demonstrated poor outcomes: UCLA Score: 21/35 (pain 6, function 4, active flexion 3, strength 3, satisfaction 5); Constant-Murley Score: 54/100 (pain 10, ADL 12, ROM 20, abduction strength 15). Through x-ray examination, anterior dislocation of the left shoulder joint was observed (Figures 2A,B). CT scan revealed a Hill-Sachs lesion in the left humeral head, pseudo-glenoid formation below the coracoid process, and a bony protuberance on the glenoid (Figures 2C,D). The MRI examination revealed a Bankart lesion, with marked thinning of the supraspinatus, infraspinatus, subscapularis, and teres minor muscles (Figures 2E,F). The deltoid muscle also showed atrophy compared to the contralateral side.

**Figure 3:**
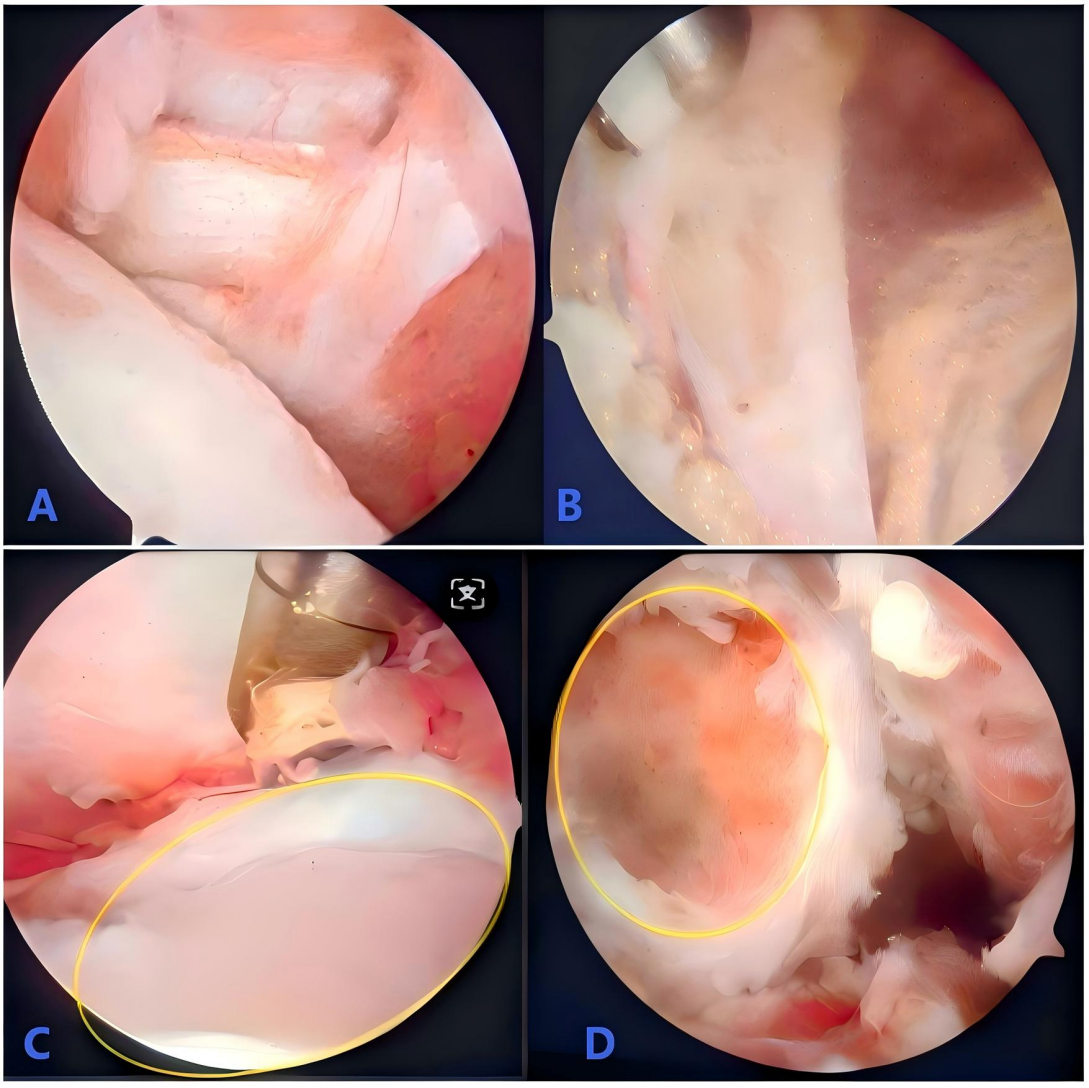
**(A)** arthroscopy shows the adhesive band of the shoulder joint. **(B)** Arthroscopy releases the adhesive band. **(C)** Arthroscopic examination revealed an empty glenoid cavity (marked by yellow circle) due to the humeral head dislocated from the glenoid. **(D)** Intraoperative image of arthroscopic glenoid reconstruction (marked by yellow circle).

Given the patient's young age, female gender, and desire for both cosmetic improvement and functional restoration of the shoulder joint, following surgical discussion and evaluation, we performed the arthroscopic release combined with Latarjet procedure on April 24, 2024. Due to the locked anterior dislocation of the left shoulder joint which made direct manual reduction under general anesthesia unfeasible, we needed to perform an arthroscopic joint release prior to reduction. During arthroscopic exploration, the following findings were observed: anterior-inferior dislocation of the humeral head, glenoid cavity emptiness, tense state of supraspinatus, teres minor and infraspinatus muscles, disappearance of the true glenoid cartilage, loss of the glenoid fossa concavity, and the presence of bony protuberance-like hyperplasia (Figure 3C). Under arthroscopic guidance, we first entered the subacromial space to debride hyperplastic adhesive bands around the humeral head. Subsequently, we entered the glenohumeral joint to release the posterior joint capsule and abrade the hyperplastic osteophytes on the glenoid surface, restoring the glenoid to its normal slightly concave configuration (Figure 3D). Finally, we performed anterior glenoid osteoplasty to remove hyperplastic sclerotic bone from the anterior aspect of the glenoid. Following arthroscopy withdrawal, an open approach was performed to identify the coracoid and conjoined tendon. The coracoid tip with partial body was osteotomized using an ultrasonic bone scalpel and mobilized. The subscapularis muscle was split to expose the humeral head, which was anatomically reduced and temporarily fixed with a 2.0-mm Kirschner wire through the glenohumeral joint. The anteroinferior glenoid rim was freshened, and the harvested coracoid tip was precisely positioned at the anteroinferior glenoid margin. Two cannulated screws were inserted parallel to the glenoid surface for fixation. Finally, the rotator cuff was repaired and anterior shoulder joint capsule was performed to tighten.

**Figure 4:**
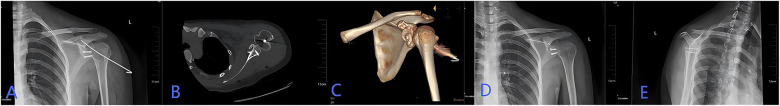
**(A)** postoperative day 2 x-ray examination showed that the shoulder joint had been reduced. **(B,C)** Postoperative day 2 CT scan revealed a well-aligned glenohumeral relationship and secure fixation of the coracoid process. **(D,E)** Follow-up x-ray examination at 3 weeks postoperatively revealed that the glenohumeral joint alignment remained consistent with previous following the removal of Kirschner wire.

Postoperative x-ray and CT scan follow-ups revealed satisfactory reduction and fixation of the left shoulder joint (Figures 4A–C). Two weeks after surgery, the Kirschner wire was removed, and the patient was instructed to begin left shoulder joint functional exercises. The three-week postoperative follow-up shoulder x-ray examination revealed that the Kirschner wire had been removed, with cannulated screws demonstrating secure fixation and the glenohumeral joint maintaining proper alignment (Figures 4D,E).

During the early postoperative period, the patient demonstrated significant limitation in left shoulder mobility, and the VAS score was 5. At the 3-week postoperative mark, the left shoulder exhibited forward flexion was 60°, abduction was 70°, VAS score was 4, UCLA score was 13, and Constant-Murley score was 44. By 6 weeks post-surgery, the left shoulder exhibited forward flexion was 90°, external rotation was 20°, VAS score was 3, UCLA score was 21, Constant-Murley score was 62,with internal rotation at the side reaching the L2 vertebral level (Figure 1B). At the 10-week postoperative follow-up visits, the left shoulder exhibited forward flexion was 110°, abduction surpassing was 90°，external rotation was 45°, VAS score was 3, UCLA score was 26, Constant-Murley score was 73,with internal rotation at the side reaching the L1 vertebral level (Figure 1C). At the 1-year postoperative follow-up, the patient demonstrated anterior flexion exceeding 150°, abduction surpassing 150°, external rotation over 60°, internal rotation at the side reaching T7 vertebral level (Figure 1D), VAS score was 0, with UCLA score of 34 points and Constant-Murley score of 94 points. After surgery, the patient resumed his daily life and work.

## Discussion

The shoulder joint, being the most mobile joint in the body, sacrifices stability for its range of motion, making shoulder dislocations the most common type of joint dislocation. It is generally accepted that a shoulder dislocation is defined as chronic when it exceeds three weeks (8–10). A chronic or neglected anterior shoulder dislocation is frequently associated with structural abnormalities including Bankart lesion, Hill-Sachs lesion, significant bone loss, rotator cuff tears, glenohumeral osteoarthritis, and tears of the shoulder joint capsule, etc (4–6). According to the literature, the available treatment options for chronic shoulder dislocation include closed reduction, open reduction with Kirschner wires, Bankart repair, Latarjet procedure, hemiarthroplasty, and reverse shoulder arthroplasty (11). Because severe soft tissue contracture and imbalance and closed reduction of bone defects are impossible, because these dislocations are usually locked in place, surgical treatment is the first choice for this patient (12).

The patient presented with a chronic locked anterior dislocation of the left shoulder joint complicated by Bankart and Hill-Sachs lesions. We considered performing a Latarjet procedure. First described in 1958, the Latarjet procedure involves transferring an osteotomized coracoid process to the glenoid rim to address glenoid bone defects (13). The Latarjet procedure not only reconstructs the curvature of the glenoid using the coracoid bone block, allowing for greater bone-to-bone contact, but also creates a sling effect through the conjoined tendon and inferior portion of the subscapularis. Additionally, it reinforces the anterior joint capsule by repairing the coracoacromial ligament. This “triple blocking” mechanism can effectively restore anterior shoulder stability and reduce the recurrence rate of dislocations (14). Intraoperative stability test confirmed that there was still slight shoulder instability after a single Latarjet operation. We used a Kirschner wire for temporary fixation according to Lubis AMT's practice (15), but there may be fretting when using one Kirschner wire, and it may be better to maintain shoulder stability by using two Kirschner wires.For patients with chronic anterior shoulder instability combined with significant Hill-Sachs lesions, both the arthroscopic Bankart-remplissage procedure and open Latarjet procedure are reliable and safe surgical options, demonstrating low and comparable recurrence rates. Some studies have shown that loss of external rotation and residual pain are more common in Bankart-remplissage surgery, while other research indicates that the Latarjet procedure is associated with a higher complication rate (4, 16). It has been suggested that compared to Bankart repair, the open Latarjet procedure demonstrates superior effectiveness in treating recurrent anterior shoulder dislocation with significant glenoid bone defects (5). Both the Bristow procedure and the Latarjet procedure belong to coracoid transfer techniques, which are commonly used surgical approaches for treating recurrent shoulder instability associated with glenoid bone defects. However, in cases of severe glenoid bone loss, the Latarjet procedure demonstrates superior stability outcomes (17). There is currently no reported treatment option for chronic locked anterior shoulder dislocation caused by epilepsy. After thorough consideration, we had decided to proceed with the Latarjet procedure for this patient.

The patient had a dislocation for over one year with significant atrophy of periarticular shoulder muscles and pseudoglenoid formation, making closed reduction of the left shoulder entirely impossible. We first performed full release of the posterior joint capsule and the surface of the rotator cuff using arthroscopy, to achieve separation between the rotator cuff and the inner surface of the deltoid muscle. During routine posterior portal access, the arthroscope entered the subacromial space with unexpected ease due to expanded subacromial dimensions from chronic anterior dislocation. Significant adhesions and synovial hyperplasia were observed in the subacromial space. While releasing the subacromial space to expose supraspinatus, infraspinatus, and teres minor muscles, the hyperplastic tissues demonstrated marked bleeding tendency. Following subacromial debridement and release, direct visualization through the intermuscular space between infraspinatus and teres minor revealed a convex-shaped hyperplastic glenoid with thinned and sclerotic articular cartilage. The humeral head showed Hill-Sachs lesion, and partial bone defects at the anterior glenoid were replaced by sclerotic hyperplastic bone. Further exploration toward the coracoid process demonstrated thinned subscapularis and pseudoglenoid formation beneath the coracoid. The posterior joint capsule was released lateral to the posterior edge of the glenoid labrum to provide adequate posterior space for humeral head reduction. Prior to reducing the glenohumeral joint, given the patient's reverse curvature of the glenoid with cartilage destruction and subchondral sclerosis, intraoperative glenoidplasty was performed to restore its normal slightly concave morphology and reestablish the ball-and-socket relationship. Due to chronic anteroinferior dislocation of the humeral head and long-term restricted shoulder function, atrophy of the rotator cuff muscles and deltoid had occurred, resulting in thinning of muscle bellies and anterior shoulder instability, which predisposed to postoperative redislocation. During intraoperative manual reduction, softening of the humeral head was noted with palpable bony depression upon digital pressure, suggestive of a posterior Hill-Sachs lesion. Performing humeral head fenestration and bone grafting risked further damage to the already compromised bone. Under circumstances of infraspinatus and teres minor atrophy due to disuse, performing the Remplissage procedure raises concerns regarding insufficient soft tissue thickness and unstable screw fixation, which may fail to meet the requirements for effective defect filling. After comprehensive evaluation, a Latarjet procedure was selected. The coracoid tip and neck were harvested with preservation of the conjoined tendon. It is reported that graft fixation using screw fixation or suture button fixation yielded similar results for arthroscopic Latarjet procedures with respect to complications and return to sports (18). Therefore, following decortication and freshening of the anterior glenoid rim, the coracoid bone block was secured into the defect site using metal cannulated screws, thereby reconstructing and augmenting the glenoid width. Intraoperative testing revealed no evidence of humeral head dislocation, and passive rotation did not result in an off-track phenomenon of the humeral head.

Recurrent seizures increase the risk of postoperative dislocation (19). Before and after the operation, it is suggested that the standard antiepileptic drugs should be treated under the guidance of neurologists, and surgery should be avoided in the active period of seizures. This patient did not have seizures for more than 6 months before the operation, so we carried out the operation. Long-term oral administration of antiepileptic drugs (such as carbamazepine) may interfere with calcium metabolism and vitamin D absorption, leading to decreased bone density and osteoporosis (20). Preoperative screening of bone mineral density and bone metabolism index is helpful to evaluate bone quality. We detected that the bone is soft during operation and slightly unstable after reduction. We chose hollow screw fixation combined with temporary Kirschner wire fixation to improve the fixation strength and prevent the graft from loosening.

In our case, a Kirschner wire transfixing the humeral head and glenoid were temporarily maintained for two weeks to allow soft tissue healing around the shoulder joint. The Kirschner wire was removed two weeks postoperatively to minimize the risk of joint adhesion or potential ankylosis. The patient was subsequently instructed in shoulder rehabilitation exercises during outpatient follow-up. After one year of rehabilitation exercises, the patient regained good range of motion and muscle strength in the shoulder joint, with no complications of nerve injury and no recurrent dislocation events occurring.

## Conclusion

Chronic anterior shoulder dislocations frequently present with concomitant pathological injuries, including Bankart lesions, Hill-Sachs defects, and significant bone loss, etc. The Latarjet procedure serves as an effective surgical intervention for chronic shoulder dislocation caused by epileptic seizure, with capsular release being a prerequisite for successful joint reduction. Early postoperative functional exercises are crucial for optimal recovery of shoulder joint functionality.

## Data Availability

The raw data supporting the conclusions of this article will be made available by the authors, without undue reservation.
